# New Sequencing technologies help revealing unexpected mutations in Autosomal Dominant Hypercholesterolemia

**DOI:** 10.1038/s41598-018-20281-9

**Published:** 2018-01-31

**Authors:** Sandy Elbitar, Delia Susan-Resiga, Youmna Ghaleb, Petra El Khoury, Gina Peloso, Nathan Stitziel, Jean-Pierre Rabès, Valérie Carreau, Josée Hamelin, Ali Ben-Djoudi-Ouadda, Eric Bruckert, Catherine Boileau, Nabil G. Seidah, Mathilde Varret, Marianne Abifadel

**Affiliations:** 1 INSERM LVTS U1148, hôpital Bichat-Claude Bernard, Paris, France; 2 Laboratory of Biochemistry and Molecular Therapeutics, Faculty of Pharmacy, Pôle Technologie- Santé, Saint-Joseph University, Beirut, Lebanon; 3 Paris Diderot University, Paris, France; 4 Laboratory of Biochemical Neuroendocrinology, Institut de Recherches Cliniques de Montréal, Affiliated to the Université de Montréal, Montréal, Québec, H2W1R7 Canada; 5 Department of Biostatistics, School of Public Health, Boston University, Boston, MA 02216 USA; 6 Division of Cardiology, Department of Medicine; Department of Genetics, McDonnell Genome Institute, Washington University School of Medicine, Saint Louis, MO USA; 7 Assistance Publique-Hôpitaux de Paris, HUPIFO, hôpital Ambroise-Paré, Laboratoire de Biochimie et de Génétique Moléculaire, Boulogne-Billancourt et UVSQ, UFR des Sciences de la Santé Simone Veil, Montigny-le-Bretonneux, France; 8 Assistance Publique-Hôpitaux de Paris, Endocrinology and Nutrition Department, Human Research Nutrition Center, Pitié-Salpêtrière Hospital, F-75013 Paris, France; 9 Service de Génétique, hôpital Bichat-Claude Bernard, Paris, France

## Abstract

Autosomal dominant hypercholesterolemia (ADH) is characterized by elevated LDL-C levels leading to coronary heart disease. Four genes are implicated in ADH: *LDLR*, *APOB*, *PCSK9* and *APOE*. Our aim was to identify new mutations in known genes, or in new genes implicated in ADH. Thirteen French families with ADH were recruited and studied by exome sequencing after exclusion, in their probands, of mutations in the *LDLR*, *PCSK9* and *APOE* genes and fragments of exons 26 and 29 of *APOB* gene. We identified in one family a p.Arg50Gln mutation in the *APOB* gene, which occurs in a region not usually associated with ADH. Segregation and *in-silico* analysis suggested that this mutation is disease causing in the family. We identified in another family with the p.Ala3396Thr mutation of *APOB*, one patient with a severe phenotype carrying also a mutation in *PCSK9*: p.Arg96Cys. This is the first compound heterozygote reported with a mutation in *APOB* and *PCSK9*. Functional studies proved that the p.Arg96Cys mutation leads to increased LDL receptor degradation. This work shows that Next-Generation Sequencing (exome, genome or targeted sequencing) are powerful tools to find new mutations and identify compound heterozygotes, which will lead to better diagnosis and treatment of ADH.

## Introduction

Hypercholesterolemia is a major risk factor for atherosclerosis and its premature cardiovascular complications. Autosomal dominant hypercholesterolemia (ADH) (MIM # 143890) is one of the most common monogenic disorders. It is caused by mutations in genes encoding key proteins involved in the LDL receptor (LDLR) endocytic and recycling pathways, causing decreased cellular uptake of LDL and increased plasma LDL-cholesterol (LDL-C) concentrations. Elevated plasma LDL-C levels give rise to tendon and skin xanthomas, arcus cornea, and vascular deposits, leading to progressive and premature atherosclerosis and coronary heart disease (CHD)^1^. Four genes are known to be implicated in the disease: *LDLR*, the gene encoding the low-density lipoprotein receptor^2^; *APOB*, which encodes the apolipoprotein B^3^, the ligand of the LDL receptor; *PCSK9* (Proprotein convertase subtilisin kexin 9)^4^, which encodes a serine protease^5^ that plays a role in the degradation of the LDLR independently of its catalytic activity^6^; and *APOE*^7^. Mutations in LDL Receptor Adaptor protein 1 (*LDLRAP1*) gene are responsible of the recessive form of the disease^8^.

The prevalence of ADH is approximately estimated to 1/217 in Northern Europe^9,10^ but varies geographically from a region to another and is higher in some populations due to a founder effect^11^. Recent studies showed that ADH is under-diagnosed and undertreated in the general population^12^ and the probability of identifying a genetic component in individuals with hypercholesterolemia increases when LDL-C levels increased^10^.

The respective contribution of each known gene to ADH slightly varies from one country to another. A study in a French cohort, which included probands and families with hypercholesterolemia recruited through the French Research Network for ADH from several regions of France, showed that the *LDLR* gene is implicated in 73.9% of the cases, while mutations in *APOB* and *PCSK9* are responsible of 6.6% and 0.7% of the cases respectively. Nevertheless, in 18.8% of the ADH probands studied, no mutation was found^13^.

Genetic diagnosis of ADH was generally performed by direct DNA sequencing or a combination of direct sequencing with the multiplex ligation-dependent probe amplification (MLPA) to detect large insertion or deletion mutations in genes known to be implicated in the disease. Concerning the *APOB* gene, the p.Arg3527Gln, also known as APOB3527 or APOB3500, is the first and most common ADH-related mutation in *APOB* reported in late 1980s^3^. It is responsible alone for more than 95% of Familial Defective apolipoprotein B cases (FDB)^14^ (MIM# 144010). A few other mutations leading to hypercholesterolemia were described in the following years and were all located in a specific region of the *APOB* gene. Therefore, this gene was not classically entirely studied in ADH by Sanger sequencing and routinely only a fragment of exon 26 and another of exon 29 are analyzed, covering the regions where the functional mutations causing hypercholesterolemia have been described^15,16^.

Targeted next-generation sequencing (NGS) panel is now currently used to screen for ADH-causing mutations^10,17^, while exome sequencing (targeted sequencing of all protein-coding regions of the genome) is used to identify mutations in new genes. This approach, now slowly substituted by whole genome sequencing, has emerged as an effective tool for gene discovery in families with suspected monogenic disorders^18^.

The aim of our study was to investigate the genetic causes of ADH in French probands. After the exclusion in 127 ADH probands, of mutations in the *LDLR*, *APOE* and *PCSK9* genes and fragments of exons 26 and 29 of *APOB*, thirteen ADH families were recruited. Two or three affected members of each family were studied by exome sequencing in order to identify new mutations in known genes or in new genes, which will eventually lead to a better understanding of the mechanism of this disease.

## Results

### A double heterozygous patient with a mutation in *APOB* and *PCSK9* in HC138 family

**Figure 1:**
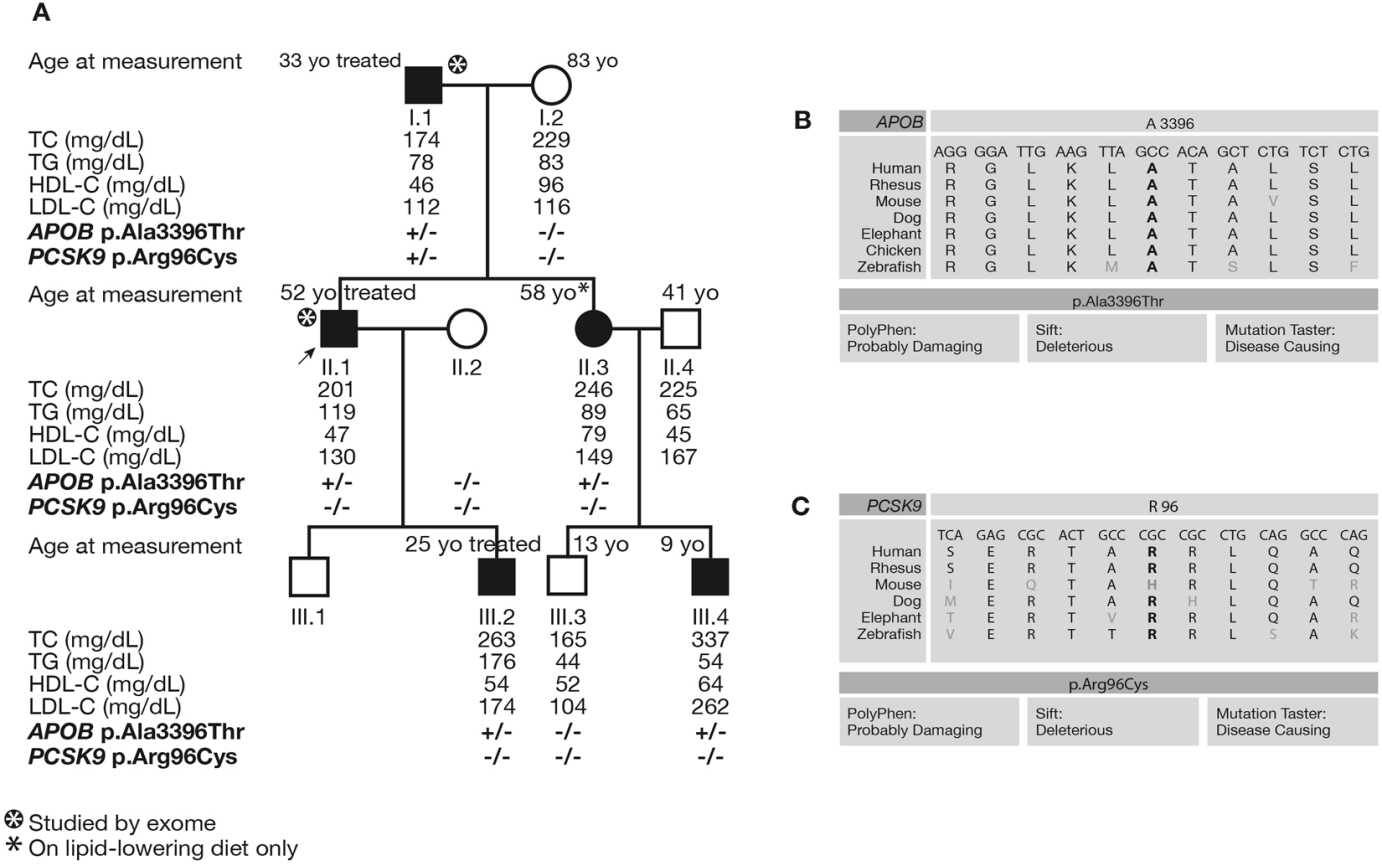
Pedigree of family HC138 with status of the p.Ala3396Thr mutation of *APOB* and the p.Arg96Cys of *PCSK9* for each patient. (**A**) Lipid levels are given when available in mg/dL with the age at clinical measurement. The −/− indicates the absence of the mutation while +/− indicates the heterozygous carriers. The asterisk shows the patients studied by exome sequencing. (**B–C**) Conservation of the alanine at position 3396 of APOB and the arginine at position 96 of PCSK9 between different species. *In*-*silico* prediction analysis of both mutations using Polyphen, Sift, and Mutation taster tools.

Exome sequencing was used to investigate the genetic cause of ADH in the HC138 family using DNA from patients I.1 and II.1 (Fig. 1). The proband II.1 had a history of arcus cornea with high levels of total-cholesterol (201 mg/dL) and LDL-C (130 mg/dL) despite treatment by rosuvastatin 10 mg and Ezetimibe. His father I.1 suffered from myocardial infarction at the age of 50. He also presented arcus cornea and had a very high level of total-cholesterol reaching 440 mg/dL before he started statin treatment. The familial autosomal dominant transmission was confirmed by the recruitment of other members of the family. Details of their clinical measurements are given in Fig. 1A.

Exome sequencing of the two patients allowed the identification of the c.10186 G > A mutation in exon 26 of the *APOB* gene, which led to the substitution p.Ala3396Thr as previously described^19^. This mutation was then confirmed by Sanger sequencing. It segregated with the disease in the family with a penetrance of 100% and with no phenocopy. It was not reported in the different databases: Exome Variant Server (evs.gs.washington.edu/EVS/), dbSNP (ncbi.nlm.nih.gov/SNP/), and gnomAD browser (gnomad.broadinstitute.org/). The *in-silico* bioinformatics tools predicted that it is disease causing, and showed a high conservation of the alanine at position 3396 among different species (Fig. 1B).

Furthermore, exome sequencing analysis showed in patient I.1 a variation in exon 2 of *PCSK9*: c.286 C > T leading to the p.Arg96Cys substitution. This variant was confirmed by Sanger sequencing. It was not carried by any other member of the family (Fig. 1A). The p.Arg96Cys (rs185392267) is reported with a very low frequency of 2.166e-5 (only 6 times over 277054 in gnomAD browser). The arginine at position 96 is highly conserved among species and its replacement by a cysteine is predicted to be pathologic by the *in silico* prediction tools (Fig. 1C).

### Characterization of the p.Arg96Cys *PCSK9* mutation

**Figure 2:**
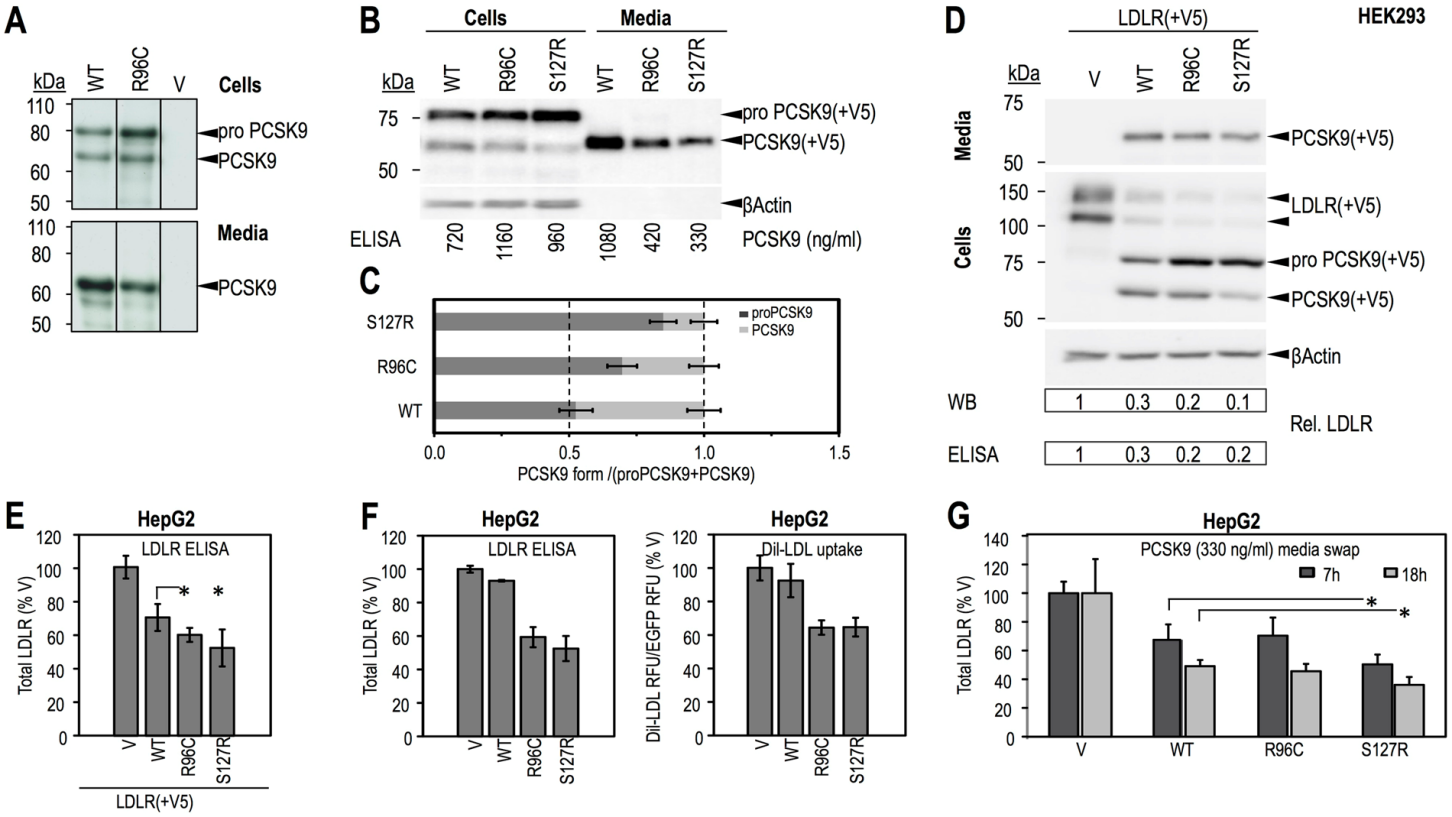
Cell-based functional characterization of PCSK9 wild-type (WT) and p.Arg96Cys (R96C). HEK293 cells were transiently transfected with V5-tagged PCSK9-WT (WT), PCSK9-R96C (R96C) or empty vector (V) (**A**), or gain of function PCSK9-S127R (S127R) (positive control) (**B** and **C**) and analyzed for PCSK9 cellular expression, zymogen processing and secretion of mature protein. (**A**) Biosynthetic analysis; 48 h post-transfection cells were pulsed-labeled with [^35^S]Met/Cys for 3 h, followed by anti-V5 immunoprecipitation, SDS-PAGE and autoradiography. (**B**) WB analysis using anti V5-HRP. Levels of total cellular and secreted PCSK9 were quantified by ELISA and the concentrations are listed. The bands corresponding to proPCSK9 and PCSK9 in (**B**) were quantified, their values normalized to β−Actin and ratios of the value of each form to the sum of the two forms were graphed in (**C**). V5-tagged LDLR was co-transfected with V5-tagged PCSK9-WT, PCSK9-R96C, PCSK9-S127R or empty vector, as control, in HEK293 cells (**D**) or HepG2 cells (**E**). Transfected HEK293 cells were analyzed by WB using anti V5-HRP (**D**). Total cellular levels of LDLR were quantified from the WB and by ELISA for HEK293 cells (**D**) or by ELISA only for HepG2 cells (**E**) and were normalized to values of the control. (**F**) HepG2 cells were transiently transfected with V5-tagged PCSK9-WT, PCSK9-R96C, PCSK9-S127R or empty vector, as control, and the total cellular endogenous levels of LDLR were quantified by ELISA (left). Dil-LDL uptake (right) was measured over 2 h. Measured values are reported as % control. (**G**) HepG2 cells were incubated for 7 h or 18 h with conditioned media from HEK293 cells (produced, illustrated and quantified in **B**): no PCSK9 control-media (V) or PCSK9-media (330 ng/ml), WT, R96C or S127R, and analyzed by ELISA for total cellular LDLR. Data are representative of two independent experiments performed at least in duplicate, with the exception of (**F**), where LDLR-ELISA was completed for one experiment performed in triplicate, while Dil-LDL uptake was measured in one experiment performed in 16 independent replicates per condition. Quantifications are averages ± SD. *p < 0.05; **p < 0.01; ***p < 0.001 (t-test). Full-length blots of Fig. 2A,B and D are presented in Supplementary Figure 2, where only the lanes selected in Fig. 2 are labeled.

Functional analyses were performed to study the effect of the p.Arg96Cys *PCSK9* mutation on PCSK9 maturation and on PCSK9-mediated LDLR degradation. PCSK9-WT and p.Ser127Arg (S127R), a well-characterized gain-of-function (GOF) mutation, were used as controls. Cell-based functional characterizations of wild-type (WT) PCSK9 and mutants R96C and S127R in HEK293 and HepG2 cells are shown in Fig. 2. Biosynthetic analysis (Fig. 2A), Western Blot (WB) (Fig. 2B) and Elisa (Fig. 2C) analyses for PCSK9 showed that the p.Arg96Cys mutation results in increased cellular levels of total PCSK9 (~60%), reduced processing of proPCSK9 zymogen to PCSK9 (by ~30%), and decreased PCSK9 secretion (by ~60%) relative to WT protein. Overexpression of PCSK9-R96C results in significantly increased LDLR degradation compared to PCSK9-WT. This was confirmed by WB and by Elisa assays for total overexpressed LDLR after V5-tagged LDLR was co-transfected in HEK293 cells with V5-tagged PCSK9-WT (WT), PCSK9-R96C (R96C), PCSK9-S127R (S127R) or empty vector (V), as control (Fig. 2D), or in HepG2 cells (Fig. 2E). Similar results were obtained for endogenous LDLR in HepG2 cells after overexpression of WT and mutants PCSK9 (Fig. 2F). Measurement of Dil-LDL uptake under these conditions (Fig. 2F, right panel), showed that R96C PCSK9, like the GOF S127R, was significantly more potent than WT PCSK9 in reducing LDL uptake. However, when added extracellularly to HepG2 cells for short or long times, PCSK9-R96C degraded endogenous LDLR to the same extent as PCSK9-WT (Fig. 2G).

### A new mutation p.Arg50Gln in *APOB*, causing ADH

**Figure 3:**
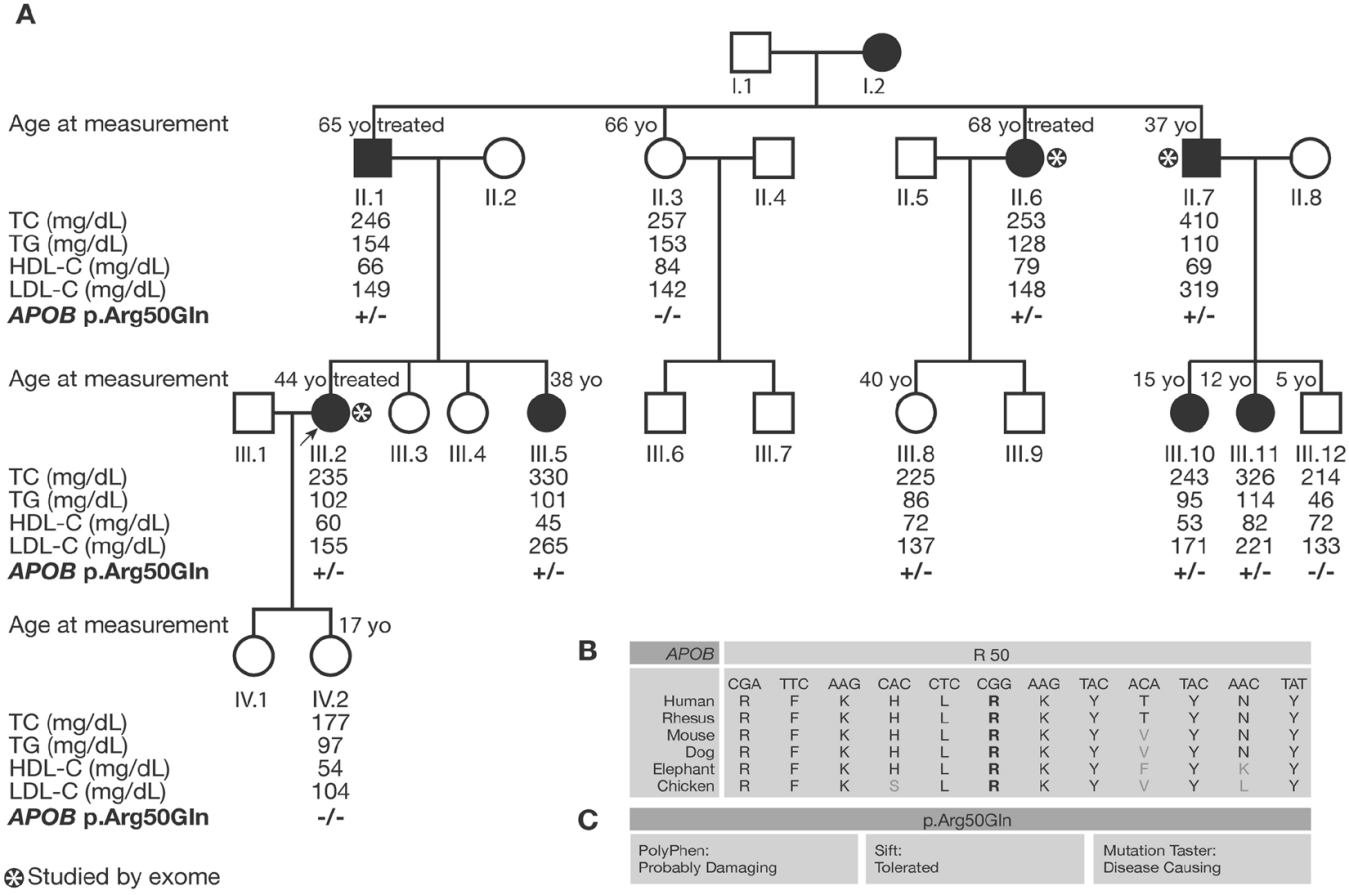
Segregation analysis of the p.Arg50Gln mutation of *APOB in* family HC706: (**A**) Lipid levels are given when available in mg/dL with the age of the patients at clinical measurement. The −/− indicates absence of the mutation while +/− indicates heterozygous carriers. The asterisk shows the patients studied by exome sequencing. (**B**) The arginine at position 50 of APOB is a conserved amino acid between different species. (**C**) This mutation is predicted to be probably damaging by Polyphen, tolerated by Sift, and disease causing by mutation taster when studied *in-silico*.

Furthermore, exome sequencing was performed in the HC706 family using DNA from II.6, II.7 and III.2 affected individuals (with asterisk in Fig. 3). The female proband (III.2) presented high levels of total cholesterol (235 mg/dL) and LDL-C (155 mg/dL) at the age of 44 even after treatment with rosuvastatin 20 mg and Ezetimibe. Family history indicated that her 65 years-old father (II.1) had a level of total cholesterol of 300 mg/dL before treatment, and kept high levels of total cholesterol (246 mg/dL) and LDL-C (149 mg/dL) at 65 years old under treatment. Interestingly, a paternal aunt (II.6) also had a history of elevated level of total cholesterol (315 mg/dL before treatment) that reached 253 mg/dL on statin treatment with a LDL-C level of 148 mg/dL at the age of 68. Additionally, a paternal uncle (II.7) presented a level of total cholesterol of 410 mg/dL and LDL-C level of 319 mg/dL at the age of 37 before starting statin treatment. Recruitment of other family members and further investigations confirmed the autosomal dominant trait. Lipid measurements of the different members of the family before treatment (when available) or after are provided in Fig. 3A.

A substitution c.149 G > A in exon 3 in the *APOB* gene causing a missense variant p.Arg50Gln was identified in the three affected members studied by exome sequencing. Sanger sequencing was used to validate the variation and showed that the variation segregated with the disease in the family with a penetrance of 91% and absence of phenocopy. Only one case of incomplete penetrance was detected over the eleven studied members of the family for whom DNA were available: individual III.8 who carries the variation but does not present high lipid levels as depicted in Fig. 3. This variant is not reported in the databases. The arginine residue at position 50 is a conserved amino acid in different species (Fig. 3B). The p.Arg50Gln variant is predicted to be damaging by bioinformatics tools such as Sift, mutation taster and Polyphen-2 as shown in Fig. 3C.

Subsequently, we analyzed by Sanger sequencing the DNA of 127 French probands with hypercholesterolemia in whom we excluded the known mutations in *LDLR*, *APOE* and *PCSK9* genes and fragments of exons 26 and 29 of *APOB*, to look for the p.Arg50Gln variation in the *APOB* gene. None of those probands carried this variation.

## Discussion

**Figure 4:**
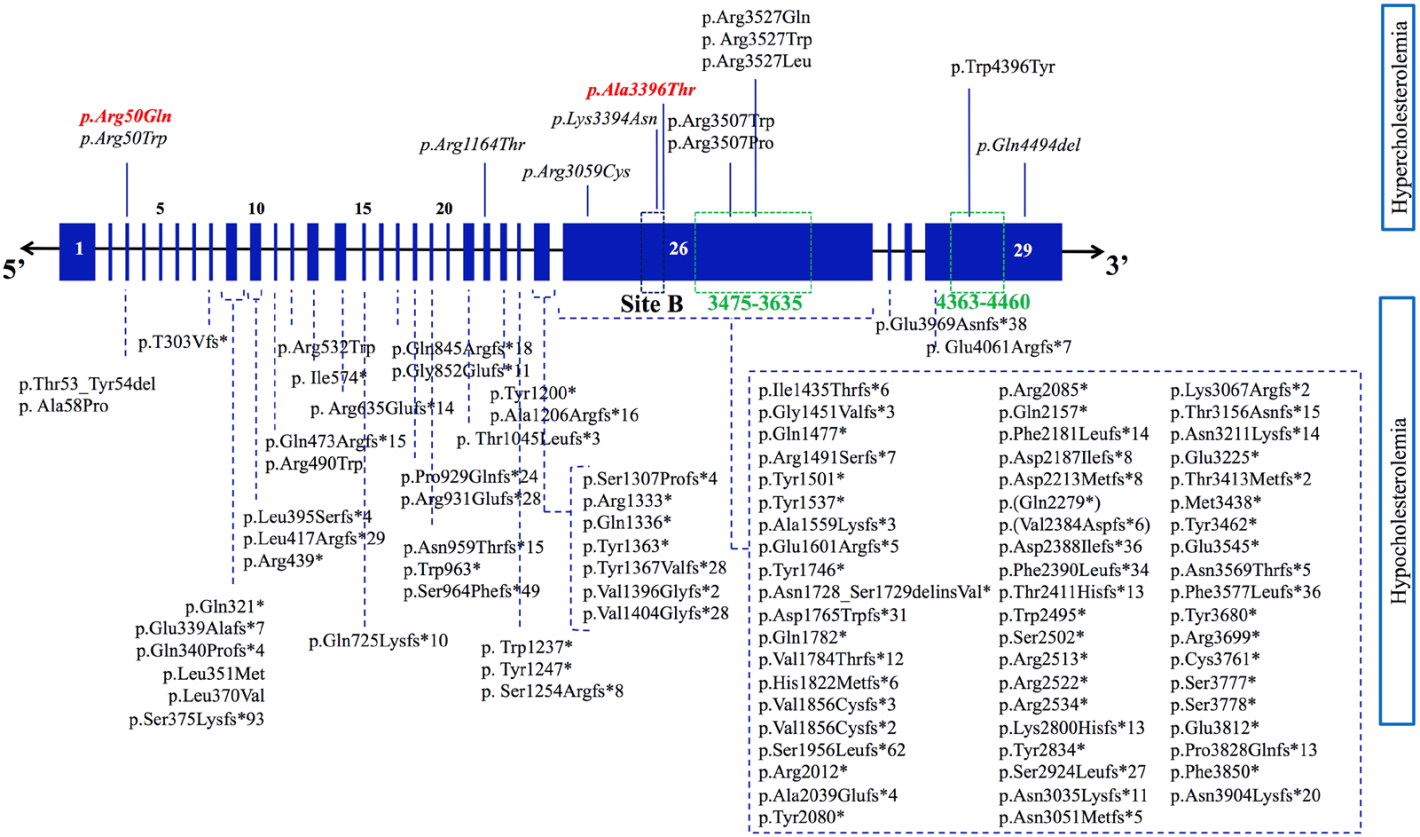
Different mutations reported in *APOB*, causing hypercholesterolemia (FDB) and hypocholesterolemia (FHBL). The *APOB* gene is constituted of 29 exons. The binding site for the LDL receptor originally described as site B is formed primarily by residues 3386–3396 (anciently known by 3359–3369). Regions 3475–3635 and 4363–4460 are the ones we classically sequence when looking for *APOB* mutations in ADH. Mutations causing familial hypocholesterolemia (FHBL) are distributed on the entire coding-region, and they are mostly nonsense, frameshift, or splicing variants. Few mutations causing hypercholesterolemia (FDB) are described in a particular region of *APOB*, the p.Arg3527Gln mutation being the most frequent one. Some others have been recently reported to cause ADH outside the classical regions of *APOB*, shown in italic; the p.Arg50Gln and p.Ala3396Thr are detailed in this article and highlighted in bold.

In this study, we show that new sequencing technologies like exome sequencing are powerful tools to find new mutations in genes already known to be implicated in ADH, *APOB* in particular, especially because the Sanger sequencing strategies classically performed in ADH genetic diagnosis do not target the entire region of *APOB*. Indeed, we identified by exome sequencing in 2 unrelated French ADH families, 2 new mutations in the *APOB* gene, p.Arg50Gln and p.Ala3396Thr which are not located in the regions of exons 26 and 29 routinely investigated using Sanger sequencing when searching for *APOB* mutations implicated in ADH. In fact, direct Sanger sequencing has been classically targeting only these particular regions due to the size of the apoB (4536 amino acids) and because the previously described functional ADH mutations are located in these specific regions of the gene^15,16^ (Fig. 4). Although numerous other mutations of *APOB* were reported in its entire coding-region, these mutations are known to cause familial hypocholesterolemia (hypobetalipoproteinemia or FHBL) and are mostly nonsense, frameshift, or splicing variants that lead to various C-terminally truncated apoB species^20^ (Fig. 4). As for FDB, the most common mutation associated with an ADH phenotype is the p.Arg3527Gln carried by approximately 0.1% of Northern Europeans and US Caucasians. In addition, the p.Arg3527Trp variant is known to make a significant contribution to familial hypercholesterolemia among East Asians^21^. Few *APOB* mutations were proven to be pathogenic in ADH: p.Arg3507Trp, p.Arg3527Gln, and p.Trp4396Tyr^15,22^. Despite the fact that these mutations are not directly involved in the binding to the LDLR segment that concerns residues 3386 to 3396^23^ (Fig. 4), they might destabilize the apoB–LDL receptor interaction by altering some critical residues that are crucial for apoB–LDL receptor affinity^24^, which result in a defective receptor-binding^23,25^. Interestingly, the p.Ala3396Thr mutation that we identified recently^19^ occurs in exon 26 of *APOB* and is located in the LDLR binding site B of apoB (Fig. 4). Consequently, this mutation might lead to a defective apoB-LDLR binding and an abnormal LDL internalization. Its good segregation with the disease in the family along with its localization in an important region for the normal apoB function are strong evidences for its pathogenic effect.

Add to that, the p.Arg50Gln mutation is new and occurs in exon 3 of *APOB*, a region not routinely analyzed in ADH. Several arguments are in favor of a mutation responsible of the ADH phenotype in this family: (1) it showed good segregation with the disease in the family, (2) *in-silico* prediction tools are in favor of its deleterious effect at the protein level, (3) this variant was never reported before, and thus its rare incidence is in favor of a mutation rather than a polymorphism and (4) exome sequencing revealed no other interesting genetic event that could be responsible for the ADH phenotype in this family. Furthermore, another ADH causing mutation at the same position, the p.Arg50Trp, has been reported in another ADH family^26^ and was confirmed by exome sequencing in the UK10K project^27^. Thomas *et al*. showed that the p.Arg50Trp variant of *APOB* accumulates in the circulation of affected carriers, which suggest its defective hepatic uptake. In fact, exon 3 forms a small part of the first domain (βα1), which is predicted to direct hepatic assembly of lipoprotein molecules, as well as to affect the interaction of LDL particles with lipases and macrophage scavenger receptors. Nevertheless, detailed information on the specific role of exon 3 in these processes is not available^26^.

Furthermore, a recent study identified 2 novel *APOB* mutations, p.Arg3059Cys and p.Lys3394Asn, both associated with a significant decrease in binding to the LDL receptor, despite having a low penetrance when studying the co-segregation in the families^16^. Another study proved that p.Arg1164Thr and p.Gln4494del of *APOB* presented a 40% decrease in internalization in lymphocytes and HepG2 cells, very similar to APOB3527^15^ even though they didn’t show complete penetrance. These results suggest that *APOB* can carry more ADH causing mutations outside of the classically studied regions, which means that all regions of the *APOB* should be investigated when diagnosing ADH. The arginine at position 50 seems to be crucial for a normal cholesterol metabolism since 2 different mutations causing ADH have been identified at this same position. Interestingly, a recent study suggested that PCSK9 could bind to amino acid sequences within the N-terminal region of apoB^28^. Whether or not *APOB* mutations in this region might affect its interaction with other proteins such as PCSK9 for instance are still to be uncovered.

Besides the p.Ala3396Thr identified in the HC138 family, the analyses of the variants observed in one of the members of this family in whom exome sequencing has been performed, allowed the identification of a new mutation in exon 2 of *PCSK9*: p.Arg96Cys. This patient carrying the 2 mutations in *APOB* and *PCSK9* showed a severe phenotype with a maximal level of 440 mg/dL of total cholesterol before statin treatment. He also suffered from a myocardial infarction at the age of 50. The p.Arg96Cys mutation of *PCSK9* has been recently reported to be responsible alone for the ADH phenotype in 3 patients from Denmark^14^, with mean untreated values of total cholesterol of 271.5 ± 46.0 mg/dL and LDL-C of 191.4 ± 34.4 mg/dL. Interestingly, 2 of those 3 patients presented coronary artery disease (CAD)^14^. Our functional studies performed herein demonstrated that the p.Arg96Cys mutation is a new GOF mutation of *PCSK9*, which alone may be responsible for the ADH phenotype. Although synthesized in cells at a higher level than the WT protein, the p.Arg96Cys is less secreted compared to WT. When overexpressed in cells, this GOF mutant degrades the LDLR to a higher extent than PCSK9-WT. The latter activity of PCSK9-R96C correlated with a decreased Dil-LDL uptake in HepG2 cells, an effect similar to that observed with the PCSK9-S127R GOF mutant. Interestingly, when added extracellularly to HepG2 cells, PCSK9-R96C degraded endogenous LDLR to the same extent as PCSK9-WT, whereas the GOF PCSK9-S127R displayed a significantly higher activity toward the LDLR. Collectively, our cell-based data suggest that PCSK9-R96C is a GOF mutant that better interacts with LDLR intracellularly (intracellular pathway)^29^, but similarly at the cell surface (extracellular pathway). In the liver, the extracellular pathway is predominant, as wild type PCSK9 acts mostly extracellularly on hepatocytes. However, the presence of a mutation on one allele of the *PCSK9* gene could result in a shift of the prevalence of one pathway over the other, as was also reported for the LDLR-R410S mutation^30^. Thus, genetic evidence together with the cell-based functional characterization of PCSK9-R96C compared to WT and S127R are strong enough to conclude that the p.Arg96Cys is a GOF mutation of PCSK9, which alone could cause ADH, and aggravate the phenotype when carried together with another ADH causing mutation.

Consequently, exome sequencing helped us identify, in a patient with severe ADH, a mutation in *APOB* together with a new mutation in *PCSK9*. To the best of our knowledge, this is the first report of an ADH patient carrying a mutation in both *APOB* and *PCSK9* genes concomitantly. Unfortunately, parents or other family members were not available for further investigations to rule out the possibility of a *de-novo* mutation that might have occurred in the *PCSK9* gene in this patient, and that it was not transmitted to his children. These findings demonstrate that exome sequencing can help in the diagnosis and the identification of compound heterozygotes in ADH. In fact, some studies described patients and families with both *LDLR/APOB*^31–36^, or *LDLR/PCSK9* mutations^37,38^. However, the clinical characteristics of double-heterozygous ADH patients are underreported and the diagnosis of double-heterozygous ADH can be easily missed^39^. Thus other *APOB/PCSK9* double heterozygous might exist. It is noteworthy that the molecular identification of double heterozygosity is very important for family screening and adequate ADH diagnosis and treatment of all affected carriers of the family. Furthermore, the other genetic event occurring in another gene might explain differences in phenotypes and would be important for revealing the cause of phenocopies when studying familial segregation. This underscores the necessity of fully screening the *LDLR*, *APOB, PCSK9* and *APOE* genes in all patients.

In summary, our work shows the importance of next generation sequencing technologies such as exome sequencing in identifying new mutations in the genes known to be implicated in ADH, and in revealing double heterozygous mutations, which improves familial screening and genetic counseling, as well as understanding the transmission of the disease and its severity in different members of the same family. The investigation for ADH mutations should include the entire coding regions of *LDLR*, *PCSK9*, *APOE* and *APOB*, with a particular attention to the region of the arginine at position 50 of the *APOB*. This will improve diagnosis and treatment of the disease and prevent its cardiovascular complications. Physicians, clinical biochemists and health professionals worldwide should join their effort to fight against this disease by offering the most adequate screening and diagnosis of individuals and families with ADH. Thus, genetic studies are of great importance in these extremely high-risk individuals and families. They can help implementing the most effective strategies to prevent and treat ADH, and might lead to new therapeutic class of lipid lowering drugs like it was the case with the discovery of PCSK9^40^.

## Methods

### Patients and Families

Probands and families from different cities in France were recruited by The French National Research Network on Hypercholesterolemia based on the inclusion criteria previously described^13^: LDL-C levels above the 95th percentile when compared with a sex and age-matched French population (STANISLAS cohort, B. Herbeth, G. Siest & S Visvikis-Siest^41^, personal communication), with normal levels of triglycerides and HDL-C, with an autosomal dominant transmission of hypercholesterolemia in the family. The study was performed in accordance with French bioethics regulations and all subjects gave informed consent. This study was conducted as part of trial # 05-07-06 approved by French Consultative Committee for the Protection of Person in Biomedical Research (CCPPRBs) Paris, Necker.

### Sanger Sequencing and MLPA

In all subjects, genes were studied sequentially: first, the p.Arg3527Gln mutation of the *APOB* (NM_000384.2) was looked for as previously described^42^ and then regions 3475–3635 and 4363–4460 of *APOB* were analyzed by Sanger sequencing. The promoters and the 18 exons of *LDLR* (NM_000527.4), as well as close flanking intronic sequences were amplified and sequenced. If no mutation was found, the search for a deletion/duplication of one or several *LDLR*’ exon(s) was performed with SALSA MLPA kit (P062) and data were analyzed with Coffalyser software (MRC-Holland). Finally, if no deletion/duplication was discovered, the 12 exons of *PCSK9* (NM_174936.3) and the 4 exons of *APOE* (NM_000041.3) as well as the flanking intronic regions were sequenced. Primer sequences and annealing temperatures are available upon request. Electrophoregrams were analyzed using Gensearch®, or CodonCode Alligner®.

### Exome Sequencing

The family members analyzed by exome sequencing where chosen based on DNA availability for at least 2 affected members of the family and presence of informed consent allowing for genetic studies with prioritization of phenotypic extremes when possible. These selected samples underwent exome sequencing at the Broad Institute after that the institutional review board and all participating sites approved the study protocols and all individuals who were selected for sequencing provided informed consent as previously described^19^.

### Analysis of Exome Sequencing Data

In order to find the causal mutation out of thousands of different variations obtained by the exome sequencing, we used a comprehensive framework for prioritizing variants, which is commonly used in the analysis of exome sequencing studies^43^. Starting with the total number of variants shared by individuals from the family, we excluded variants conflicting with the ADH inheritance pattern and common variants that have a frequency of ≥ 1% in the general population, by examining different databases such as dbSNP, 1000Genome, Exac and gnomAD browsers. Then, we excluded silent and non-genic variants which do not alter protein sequence since most Mendelian syndromes are caused by coding or splice site mutations that alter the protein sequence. The remaining single nucleotide variants and short insertions or deletions were considered candidates. The variants were subsequently analyzed separately by exploring those whose genes have physical protein-protein interaction with the 4 known genes of ADH, or share the same biological pathways. When an interesting variation is found, familial segregation was studied to demonstrate its possible co-segregation with the phenotype in the family.

### *In silico* Analyses

The frequency of the variations found by sequencing was estimated using different databases: Exome Variant Server (evs.gs.washington.edu/EVS/), dbSNP (ncbi.nlm.nih.gov/SNP/), and gnomAD browser (gnomad.broadinstitute.org/).

The causal effect of each new molecular event was estimated with *in silico* prediction of protein function tools: Polyphen (genetics.bwh.harvard.edu/pph), SIFT (sift.jcvi.org), MutationTaster (mutationtaster.org) using Alamut Visual version 2.7.1.

### cDNAs, cell culture and transfections

The cDNAs encoding human LDLR^30^ and human PCSK9 and its mutants^44^ were cloned in pIRES2-EGFP (Clontech Labs), a bicistronic plasmid allowing the independent expression of a fluorescent EGFP from an internal ribosome entry site (IRES) and C-terminally V5-tagged wild-type (WT) human PCSK9 or its p.Arg96Cys (R96C) mutant (identity confirmed by DNA sequencing) under the control of a CMV promoter. The WT PCSK9 and the GOF PCSK9-S127R^44^ served as control. HEK293 (human embryonic kidney-derived epithelial) and HepG2 (human hepatocellular carcinoma) cells (American Type Culture Collection, Manassas, VA) were cultured in Dulbecco’s modified Eagle medium (DMEM) (HEK293 cells) or in Eagle minimal essential medium (EMEM) (HepG2 cells) supplemented with 10% (v/v) fetal bovine serum (FBS) (Invitrogen) and were maintained at 37 °C under 5% CO_2_. HEK293 cells were seeded in poly-L-lysine (50 µg/mL) coated 12-well plates (3.5 × 10^5^ cells/well) or 10 cm plates (4 × 10^6^ cells/well) for PCSK9 media production and the following day transfected using jetPRIME (PolyPlus) and a total of 0.5 µg of cDNA or 6 µg of cDNA, respectively. 24 h post-transfection, the culturing medium was changed to serum-free and the cells were treated according to each experiment. Alternatively, for media swap experiments, HepG2 cells (3.5 × 10^5^ cells/well) were seeded in 12-well cell culture plates. 24 h later cells were starved for 24 h in serum-free media and following were incubated for 7 h or 18 h with 24 h serum-free conditioned media of HEK293 cells overexpressing human *PCSK9* (WT, S127R or R96C) (see above). The concentrations of the secreted PCSK9 into the media were measured using an *in-house* ELISA assay as previously described^45^. For Dil-LDL uptake experiments and LDLR ELISA measurements, HepG2 cells were plated in 96-well plates (0.2 × 10^5^ cells/well) (CellBind black plate with clear bottom, Corning; Cat # 3340) or 12-well plates (2 × 10^5^ cells/well), respectively. Transfections were performed at the time of seeding with 0.125 µg of cDNA (96-well plate) or 1 µg of cDNA (12-well plate) and using FuGENE^®^ HD (Promega). 24 h post-seeding and -transfection, the cells were starved for 24 h in serum-free media and treated according to each experiment.

### Biosynthetic Analyses

48 h post-transfection, HEK293 cells were washed and pulse-labeled for 3 h with 250 µCi/ml [^35^S]Met/Cys (PerkinElmer Life Sciences)^44^. Following 2 washes with ice-cold PBS, the cells were lysed in modified radioimmune precipitation assay buffer (150 mM NaCl, 50 mM Tris-HCl, pH 7.5; 1% Nonidet P-40; 0.5% sodium deoxycholate; 0.1% SDS) and protease inhibitor mixture (Roche Applied Science). Cell lysates and media were immunoprecipitated with monoclonal V5-Ab (1:500; Invitrogen) and immunoprecipitates were resolved by SDS-PAGE (8% Tris-Tricine gels) followed by autoradiography (2 h at −80 °C).

### Human LDLR ELISA in cell lysates

Following the treatments specific to each experiment, HEK293 or HepG2 cells were washed twice with ice-cold PBS and lysed on ice with ice-cold, non-denaturing cell lysis buffer (20 mM Tris-HCl, pH 8, 137 mM NaCl, 2 mM Na_2_EDTA, 1% NP-40, 10% glycerol, 4% protease inhibitor cocktail without EDTA) for 40 min, with gentle rotation. Cell lysates were cleared by centrifuging for 12 min at 15,000xg at 4 °C. The supernatants corresponding to the non-denatured cell lysates were saved and subjected to measurement of total human LDLR protein levels (human LDLR DuoSet ELISA Development kit, DY218; R&D Systems) and of total protein (Bio-Rad DC Protein assay), following the manufacturers’ protocol. The optical densities of the colored products were determined using a SpectraMax *i3* plate reader (Molecular Devices). All measured LDLR concentrations (pg/mL) were corrected for total protein concentration (mg/mL). Corrected LDLR content (pg LDLR/ml total protein) is reported as % vector (pIRES-EGFP) control.

### Western Blotting

Following the incubation times and treatments specific to each experiment, cultured cells were washed and lysed and the lysates cleared, as described above. Thirty to fifty micrograms of protein were separated on 8% Tris glycine SDS-PAGE gels and transferred to a PVDF membrane. Western blotting was performed for human LDLR-V5 and human PCSK9-V5 (anti-V5-HRP, 1:5000; R96125; Invitrogen) and for β-actin (rabbit anti-β-actin, 1:5000; A2066; Sigma). After incubation with the appropriate secondary antibodies, if required, the membranes were revealed using Clarity Western ECL Substrate (Bio-Rad), imaged with a GelDoc XR^+^ instrument (Bio-Rad) and the bands of interest quantified using ImageLab 5.2.1 software (Bio-Rad).

### Dil-LDL uptake

48 h post-seeding and -transfection and after a 24 h starvation in serum-free medium, HepG2 cells were incubated for 2 h at 37 °C with 6 μg/ml Dil-LDL (Alfa Aesar; Cat # J675330) (20 μl of 36 μg/ml Dil-LDL were added to each well containing 100 μl of conditioned media). Each condition was prepared in 16 replicates. At the end of the 2 h incubation, the media was removed and the cells washed 3 times (200 μl/well) with ice-cold D-PBS no ions (Wisent). After the final wash was removed, 100 μl of PBS was added to each well and the plate was scanned (bottom read) on a SpectraMax i3 plate reader (Molecular Devices). For each well, raw Dil-LDL uptake was measured as the average fluorescence intensity (RFU) (ex: 534 nm/em: 572 nm) of 21 points equally distributed in a fill pattern. Dil-LDL uptake in each well was corrected for the total number of transfected cells (average EGFP fluorescence intensity; ex: 488 nm/ em: 513 nm) and reported as the fluorescence ratio of Dil-LDL/EGFP. Corrected Dil-LDL uptake is reported as % vector (pIRES-EGFP) control and was obtained from 16 wells.

## Electronic supplementary material

Supplementary information
