# Supplementary material for: New Sequencing technologies help revealing unexpected mutations in Autosomal Dominant Hypercholesterolemia
Source: Sci Rep. 2018 Jan 31;8:1943. doi: 10.1038/s41598-018-20281-9 (PMC5792649; doi:10.1038/s41598-018-20281-9)
Supplement: Supplementary file 1 — Supplementary information [file 41598_2018_20281_MOESM1_ESM.pdf]

New Sequencing technologies help revealing unexpected mutations in Autosomal Dominant Hypercholesterolemia

Sandy Elbitar [1,2,3], Delia Susan-Resiga[4], Youmna Ghaleb [1,2,3], Petra El Khoury [1,2], Gina Peloso[5], Nathan Stitziel[6], Jean-Pierre Rabès [1,7], Valérie Carreau[8], Josée Hamelin[4], Ali Ben- Djoudi-Ouadda[4], Eric Bruckert[8], Catherine Boileau[1,3,9], Nabil G. Seidah[4], Mathilde Varret [1,3], Marianne Abifadel* [1,2]

Figure 2A

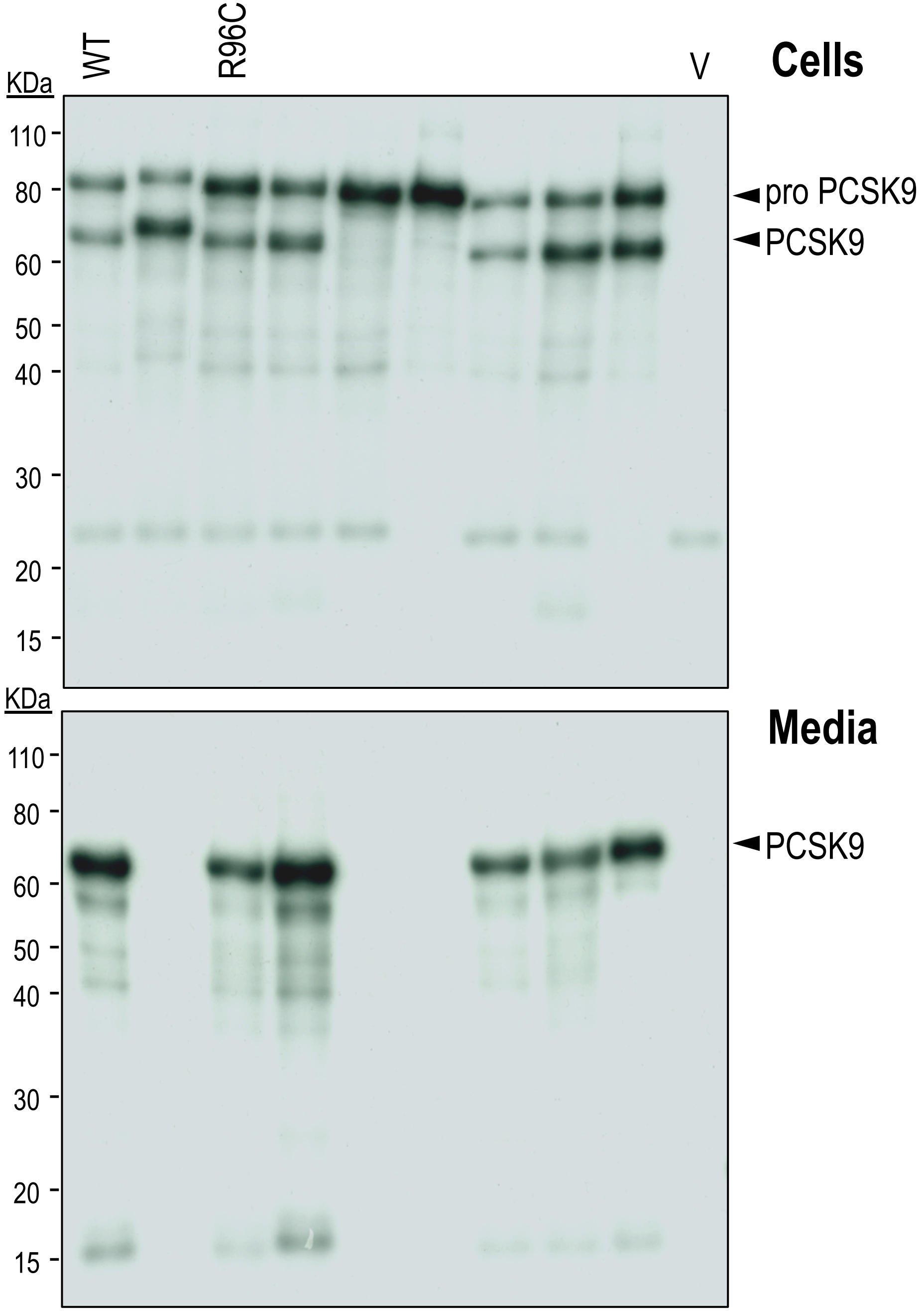

WT
R96C
V
Cells
KDa
110
80
60
50
40
30
20
15
◄pro PCSK9
◄PCSK9
Media
KDa
110
80
60
50
40
30
20
15
◄PCSK9

Figure 2B

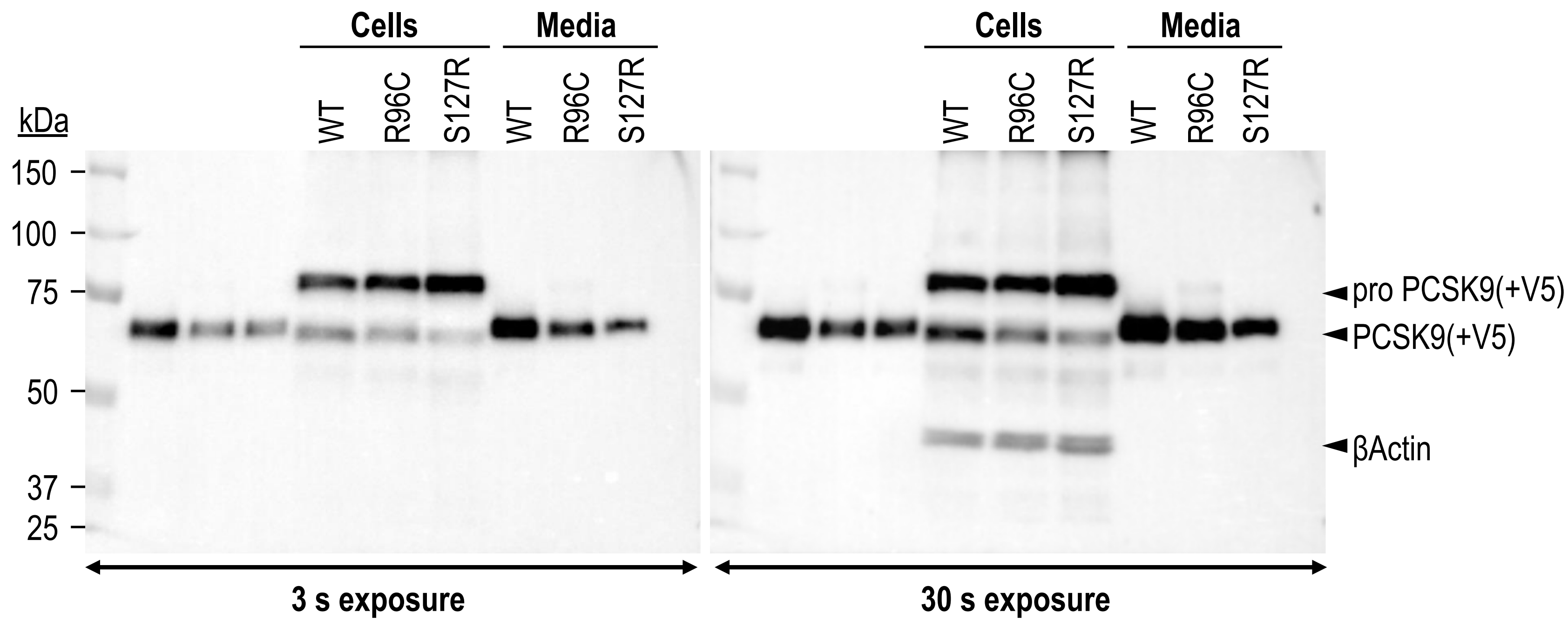

Cells
Media
WT
R96C
S127R
WT
R96C
S127R
kDa
150
100
75
50
37
25
Cells
Media
WT
R96C
S127R
WT
R96C
S127R
pro PCSK9(+V5)
PCSK9(+V5)
βActin
3 s exposure
30 s exposure

Figure 2D

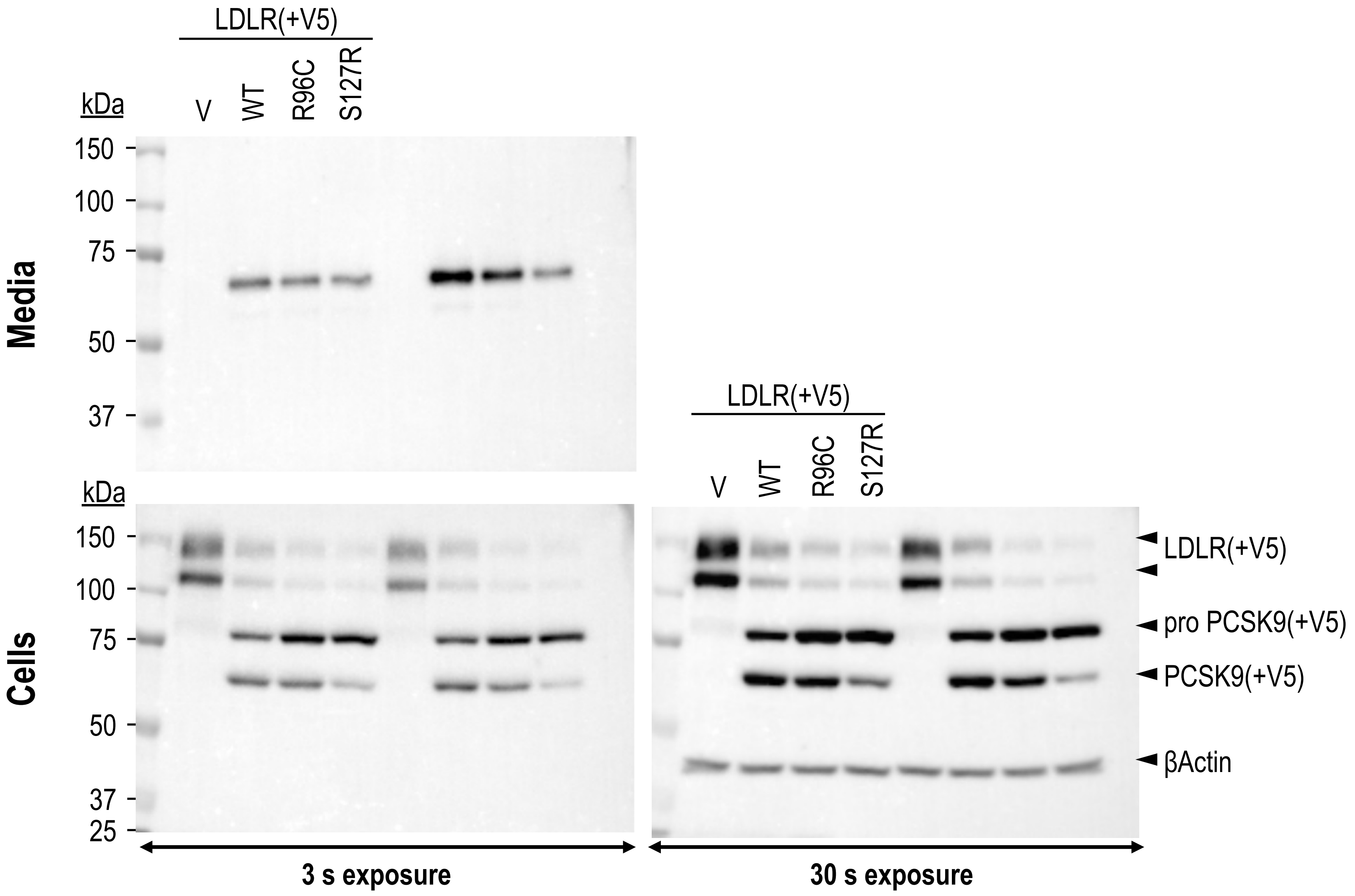
LDLR(+V5)
V
WT
R96C
S127R
kDa
150
100
75
50
37
25
Media
Cells
LDLR(+V5)
pro PCSK9(+V5)
PCSK9(+V5)
βActin
3 s exposure
30 s exposure

Supplementary Figure 2: Full-length blots of Figures 2A, 2B and 2D. Only the lanes selected in Figure 2 are labeled. 2A represents the biosynthetic analysis: 48h post-transfection cells were pulsed-labeled with [^{35}S]Met/Cys for 3h, followed by anti-V5 immunoprecipitation, SDS-PAGE and autoradiography. 2B represents WB analysis using anti V5-HRP and anti-β-Actin. The bands corresponding to proPCSK9 and PCSK9 in cells and media were quantified and their values normalized to β-Actin. In 2D, transfected HEK293 cells were analyzed by WB using anti V5-HRP after that V5-tagged LDLR was co-transfected with V5-tagged PCSK9-WT, PCSK9-R96C, PCSK9-S127R or empty vector as control and their values normalized to β-Actin.
